# Teachers’ Interpersonal Style in Physical Education: Exploring Patterns of Students’ Self-Determined Motivation and Enjoyment of Physical Activity in a Longitudinal Study

**DOI:** 10.3389/fpsyg.2018.02721

**Published:** 2019-01-10

**Authors:** Gracielle Fin, Juan Antonio Moreno-Murcia, Jaime León, Elisabeth Baretta, Rudy José Nodari Júnior

**Affiliations:** ^1^Sports Research Center (CID), Universidad Miguel Hernández de Elche, Alicante, Spain; ^2^Department of Physical Education, University of West Santa Catarina, Joaçaba, Brazil; ^3^Department of Education, University of Las Palmas de Gran Canaria, Las Palmas, Spain

**Keywords:** motivation, self-determination, physical activity, enjoyment, adolescents

## Abstract

This longitudinal study explored patterns of basic psychological needs and self-determined motivation, as well as its association with the teaching style and the physical activity enjoyment in a group of students. The sample consisted of 200 secondary education students (105 girls and 95 boys) aged 11 to 13 years (*M* = 12.65, *SD* = 0.79) at the start of the study. Students were assessed twice in a 22 month-period. Descriptive analyses were conducted between major variables at both time points, and to explore the number and nature of clusters, we relied on latent profile analysis. The statistical analyses showed three different patterns: downward, stable and upward, with different outcomes and determinants. The downward pattern was associated with more negative enjoyment and a controlling style, while the upward pattern with more positive enjoyment and autonomy support. This study, which was based on a person-centered approach, provided a bigger picture of the interplay between autonomy, competence, relatedness, self-determined motivation, autonomy/control support, and physical activity enjoyment.

## Introduction

The importance of regular exercise is indisputable in maintaining quality of life and health. Aspects related to physical exercise are particularly investigated in childhood and adolescence, since during this period a significant relationship is established between acquired behaviors and the resulting actions in adulthood (Hallal et al., 2012). Thus, a gradual increase in physical activity would be beneficial; however, a decline in these activities has been observed from adolescence onward, with high rates of physical inactivity in young people (Sousa and Hallal, 2015).

The factors that interfere in exercise adherence and a healthy lifestyle are related to motivational aspects. In this respect, the self-determination theory (SDT; Deci and Ryan, 1985, 2000), and the hierarchical model of intrinsic and extrinsic motivation (HMIEM) (Vallerand, 1997, 2001, 2007) offer an explanation of factors that promote or inhibit a healthy lifestyle, such as enjoyment during physical activity classes.

Basic psychological needs (autonomy, competence, and relatedness) and motivation are factors that have been extensively studied (Tessier et al., 2010; Cheon et al., 2014; Sparks et al., 2015). Cross-sectionals and longitudinal studies have focused on the linear relationships between autonomy, competence, relatedness or self-determined motivation and positive outcomes during physical activity classes (e.g., enjoyment, engagement or satisfaction). Similar relationships have been observed between contextual factors (e.g., teachers’ control or autonomy support) and autonomy, competence, relatedness or self-determined motivation (Lim and Wang, 2009; Cairney et al., 2012; Cheon et al., 2012).

Unfortunately, these studies only provide information on the relationship between the determinant and the outcome, such as high values on autonomy correspond to high values on enjoyment. These types of studies rely on a variable-centered approach. This approach provides a small picture of the interplay among the studied variables, however, a person-centered approach allows the study of different variable configurations whilst providing a bigger picture (Kimiecik and Horn, 2017; León and Liew, 2017). For instance, in longitudinal research we could forecast groups of students based on autonomy, competence, relatedness and self-determined motivation changes, and then test how these groups differ on the outcomes (e.g., enjoyment) and the determinants (e.g., autonomy support).

The present study addresses this gap in the literature by examining students’ patterns of autonomy, competence, relatedness and self-determined motivation changes, and how such patterns predict enjoyment/disenjoyment, and how these patterns relate to autonomy support.

### Self-Determination Theory and Basic Education

The SDT, proposed by Deci and Ryan (1985, 2017), aims to explain human behavior, based on different motivational styles, influences of the context, and interpersonal perceptions. Three basic psychological needs are related to motivation: autonomy, which is related to the level of independence and control of the choices made by an individual; competence, which refers to a person’s ability to perform a task; and relatedness, which is linked to the perception of a sense of connection with other people (Deci and Ryan, 2012).

Motivation varies from the most self-determined form, called intrinsic motivation, when the choice is personal, characterizing total autonomy in terms of the activity, which generates interest, enjoyment and satisfaction inherent to the activity (Deci and Ryan, 2000) to the least self-determined levels, which are an extrinsic motivation and amotivation (Deci and Ryan, 2000).

Satisfaction of basic psychological needs and self-determined motivation are associated with enjoyment and effort in performing activities and other positives outcomes (Deci and Ryan, 2017). For instance, Brunet et al. (2016) observed in adolescents that autonomy, competence and relatedness predicted moderate-to-vigorous-intensity physical activity 4 months later. Similarly, Richards et al. (2017) observed that self-determined motivation predicted physical activity.

Unfortunately, these longitudinal studies relied on a variable-centered approach, and thus, it is not possible to see a big picture of the interplay among the studied variables. Nevertheless, there are cross-sectional studies that relied on a person-centered approach to increase adolescents’ motivation. Most of these studies analyzed the coexistence of motivational regulations (Ullrich-French and Cox, 2009; Wang et al., 2016), but little information is available about the interplay between autonomy, competence, relatedness and self-determined motivation. To the best of our knowledge, only Kimiecik and Horn (2017) explored different clusters based on adolescents’ needs, observing three clusters based on students’ autonomy, competence, relatedness, subjective well-being and self-awareness, and perceived choice as indicators of self-determination. In this cross-sectional study, they observed three different clusters: low, moderate and high. Interestingly, students in the high cluster reported the highest levels of moderate-to-vigorous physical activity and the most healthy eating patterns.

### Interpersonal Teacher Style in Physical Education

In the educational and behavioral context, the SDT was broadened by Vallerand (1997, 2001, 2007) through the hierarchical model of intrinsic and extrinsic motivation (HMIEM). This model analyzes motivational changes (intrinsic, extrinsic and amotivation) that may occur over time, depending on three levels of social factors. The global level refers to overall motivation, which is related to family and cultural aspects developed in the first socialization processes. The contextual level refers to a specific context, such as physical activities. Finally, the situational level is influenced by the global level, and as a function of the learning styles displayed in the latter, perceptions and future styles at the contextual level may vary, in a specific situation, a particular physical exercise, for example.

During physical education classes, one of the contextual factors that can influence motivation is the teacher’s interpersonal style. Teachers offer support along a continuum, which ranges from extreme control to total support for autonomy (Tessier et al., 2010). According to Reeve (2009), supporting autonomy consists of nurturing the student’s inner motivational resources, providing basic explanations, using non-controlling language, and showing patience in allowing them the necessary time to learn at their own pace. As such, students become more involved in decision making and use an inquisitive methodology, attributing more importance to the process and encouraging effort and personal improvement (Moreno-Murcia et al., 2014).

In contrast to an interpersonal style that supports autonomy in the classroom, teachers with a controlling style ensure that activities are performed exactly according to their way of thinking, feeling and behaving. When teachers use controlling support, they make students abandon their own inner motivational resources in order to undertake activities and seek to solve problems based on the teachers’ needs (Moreno-Murcia et al., 2012).

The motor and affective experiences perceived by students during class are influenced by the teachers’ interaction and how they present class content (Jang et al., 2012; Ntoumanis et al., 2018). The teaching strategies used can affect how competent students feel to execute activities and pursue results, leading to a positive or negative change in behavior in relation to the proposed objective (Sparks et al., 2015).

Investigations on the SDT indicate that a teaching style aimed at supporting autonomy results in improved student motivation. Wang et al. (2016) observed that students in clusters characterized by the highest levels of autonomous regulation were associated with teachers with a more autonomy-supportive style.

### Physical Activity Enjoyment

A determining factor in physical education classes and physical activity in general is the feeling of joy. Studies show that self-determined motivation to engage in physical activity is influenced by perceived enjoyment and challenges that activities can generate in children and adolescents (Motl et al., 2001; Cairney et al., 2012). The pleasure experienced during physical education classes was also identified as a predictor of future physical activity in both children and adolescents (Sallis et al., 2000).

The SDT highlights the positive effect of enjoyment and the feeling of pleasure as a crucial point for physical education self-determined motivation. Perceptions of competence, autonomy, success and good relationships with others increase pleasure during physical activity and reduce negative perceptions such as boredom (Baron and Downey, 2007). Taken together, these findings suggest that autonomy, competence and relatedness may be important factors that affect the pleasure experienced during physical activity.

### The Present Study

Understanding different patterns of self-determined motivation in physical education classes may help teachers improve the quality of interactions with students, favoring and increasing positive experiences during classes (Moreno-Murcia and Sánchez-Latorre, 2016). Different researchers studied the relationship between motivational variables within the SDT, unfortunately there is lack of research focusing on patterns of longitudinal changes. Therefore, in this longitudinal study we aim to explore patterns of change of motivational variables (autonomy, competence, relatedness and self-determined motivation), and how these patterns differ on enjoyment and on teacher style (autonomy/control support).

We hypothesize the existence of different clusters and that the students in motivational clusters with more self-determination will show more pleasure in practicing physical activity and more autonomy support from their teachers. Similarly, we expect that motivational clusters with less self-determination will show less pleasure in practicing the activity and will receive more controlled classes from their teachers.

## Materials and Methods

### Participants

The sample consisted of 200 schoolchildren, 105 girls, and 95 boys, in the final grades of elementary schooling at four public schools located in the urban zone of three municipalities in Midwest Santa Catarina state, Brazil. The students were 11–13 years old (*M* = 12.65, *SD* = 0.79) at the beginning of the study. For the data analysis, the same individuals were considered for collection 1 when they were still in the sixth, seventh and eighth grades, and collection 2 were in the seventh, eighth and ninth grade, respectively.

### Measurements

#### Autonomy Support

The Learning Climate Questionnaire (LCQ; Williams and Deci, 1996) was used to determine the students’ perception of their teachers. As recommended by Núñez et al. (2012) we applied the short version, with the adaptation for Brazilian studies in Fin et al. (2017). This scale consists of 5 items preceded by the stem “My physical education teacher...,” which evaluate autonomy support (e.g., “Tries to understand how I feel before suggesting a new way of doing things”). Answers were scored on a Likert-type scale ranging from 1 (*I completely disagree*) to 7 (*I completely agree*). Since this instrument has not yet been adapted to the Brazilian educational setting, Hambleton’s back-translation method was used (Hambleton, 1996). Items were first translated into Portuguese and then translated back into English. Next, the questionnaires were applied to a small group of students to check for understanding and make any necessary corrections. Internal consistency of the scale was calculated using Cronbach’s alpha, obtaining an alpha value of 0.91. We tested the factor structure by means of a confirmatory factor analysis. All standardized loadings were between 0.661 and 0.870. Regarding the CFA, the χ^2^ value and fit indexes were: χ^2^ (199, 5) = 9.686 (*p* = 0.08), RMSEA = 0.068 [0.000, 0.133] and CFI = 0.986.

#### Control Support

The Controlling Teacher Questionnaire (CTQ), from Jang et al. (2009), was used in its modified version for physical education (Cheon et al., 2014). This scale consists of four items, preceded by “My physical education teacher is …,” which assesses teacher control during class (e.g., “Seeks/intends to control everything I do”). The answers were scored on a Likert-type scale, whose scores varied from 1 (*I completely disagree*) to 7 (*I completely agree*). The same procedure used in the previous scale was followed. Cronbach’s alpha was 0.86. All standardized loadings were between 0.270 and 0.565. Regarding the CFA, the χ^2^ value and fit indexes were: χ^2^ (199, 2) = 0.130 (*p* = 0.94), RMSEA = 0.000 [0.000, 0.035] and CFI = 0.999.

#### Basic Psychological Needs

A questionnaire was applied to assess basic psychological needs in physical education (NPBEF), adapted for Portuguese by Pires et al. (2010) from the Basic Psychological Needs in Exercise Scale (BPNESp) (Vlachopoulos and Michailidou, 2006). The questionnaire consists of 12 items encompassing three dimensions: autonomy (e.g., “I feel I do activities the way I want to”), competence (e.g., “I feel I complete class activities successfully”), and relatedness (e.g., “I feel good with my classmates”). Items are preceded by the statement “Generally, in physical education...” and are scored on a 5-point Likert scale from 1 (*I completely disagree*) to 5 (*I completely agree*). Cronbach’s alpha was 0.73, 0.71, and 0.85, respectively.

#### Self-Determined Motivation

The Perceived Locus of Causality Questionnaire (PLOCQ) (Goudas and Biddle, 1994) was used, translated into Portuguese and validated for the Brazilian population (Tenório, 2014). The questionnaire contains 20 items and is subdivided into five dimensions: intrinsic motivation, identified regulation, introjected regulation, external regulation, and amotivation. Items are preceded by the stem “I do physical education...” and are scored on a 7-point Likert scale ranging from 1 (*I completely disagree*) to 5 (*I completely agree*). Internal consistency was 0.81, 0.76, 0.76, 0.69, and 0.74, respectively. To determine the total score of self-determined motivation, the index of self-determination (IAD): (2 × intrinsic motivation + identified regulation) – [(introjected regulation + external regulation) / 2 + 2 × amotivation] (Vallerand and Rousseau, 2001) was used.

#### Physical Activity Enjoyment Scale

We applied the Physical Activity Enjoyment Scale (PACES) (Motl et al., 2001), translated by Montanha (2013), to measure the enjoyment for physical activity. The scale consists of 16 items preceded by the statement “When I am physically active...” which are divided into two groups composed of eight items each. The items of one group directly assess enjoyment, with affirmative sentences (e.g., “I enjoy it,” “It’s very pleasant,” “It gives me energy”), and the items of the other group indirectly assess enjoyment, with negative sentences (e.g., “It makes me sad,” “I dislike it,” “It’s not fun at all”). Answers were scored on a Likert-type scale, rated from 1 (*I completely disagree*) to 5 (*I completely agree*). Cronbach’s alpha was 0.91.

### Procedure

Questionnaires were administered individually during class time on two time points. The first data collection took place in March 2015 and the second in December 2016. Both evaluations had the same sample of individuals, so the data represent the answers of the students at the beginning of the 2015 school year while they were in the sixth, seventh and eighth grades. At the end of 2016, these same students were in the seventh, eighth and ninth grade, respectively. It is important to consider that the Brazilian school year begins in February and ends in December. The questionnaires were applied in the classroom under the supervision of the authors of this study. Prior authorization was requested from management staff and teachers at the schools involved in the study, as well as the parents and/or guardians of the participants. The questionnaires were answered during physical education (PE) classes under the supervision of the researcher, who explained how subjects should complete the instrument and remained available to answer any questions that might arise during the process. Completion time was approximately 35 min, and anonymity was respected to ensure sincere answers. Whilst the students answered the questionnaire, only the researchers were in the classroom. The teachers were not present during the data collection. This study was carried out in accordance with the recommendations of Human Research Ethics Committee of Unoesc/Hust with written informed consent from all subjects. All subjects gave written informed consent in accordance with the Declaration of Helsinki. The protocol was approved by the Human Research Ethics Committee of Unoesc/Hust, under protocol number 937.597 on December 19, 2014.

### Data Analyses

#### Preliminary Analyses

Descriptive analyses were conducted, including Pearson’s correlations between major variables at both time points (see Table 1). To compute variables’ indicators, we began by estimating the mean for each variable. Then, we computed the difference between each time point (T_2_ – T_1_). Finally, to ease interpretation, we standardized these values (mean = 0 and standard deviation = 1).

**Table 1:** Descriptive statistics and Pearson’s correlations.

	*M* T_1_	*M* T_2_	1	2	3	4	5	6	7
(1) Self-determination	10.061	8.175							
(2) Autonomy	3.059	3.041	0.294						
(3) Competence	3.931	3.695	0.381	0.208					
(4) Relatedness	4.071	3.898	0.190	0.120	0.182				
(5) Enjoyment +	4.002	3.525	0.614	0.198	0.369	0.167			
(6) Enjoyment –	1.625	1.834	-0.609	-0.269	-0.273	-0.129	-0.574		
(7) Autonomy Support	4.519	3.753	0.454	0.311	0.280	0.082	0.487	-0.360	
(8) Control Support	2.404	2.840	-0.220	-0.160	-0.124	-0.053	-0.007	0.172	-0.142


*All correlations were significant (p < 0.05).*

#### Latent Profile Analysis

To explore the number and nature of clusters, we relied on Latent Profile Analysis (Collins and Lanza, 2010; Berlin et al., 2014). Following the recommendations of Marsh et al. (2009) and Collins and Lanza (2010) to choose the number of clusters, we analyzed: (1) Akaike information criterion (AIC), (2) sample-size-adjusted Bayesian information criterion (SSA-BIC), (3) Parametric Bootstrapped Likelihood Ratio Test (PB-LRT), and (4) because solutions with few participants (e.g., 1 or 5% of the total sample) may not truly represent a unique cluster, we also relied on the percentage of cases in the smallest latent subgroup of each model. All of the calculations were done with Mplus 7.4 (Muthén and Muthén, 2017).

## Results

### Preliminary Analyses

Means for variables at both time points and correlations between the standardized differences scores (T_2_–T_1_) are displayed in Table 1. The means varied between 1.625 (T_1_ Negative Enjoyment) and 10.061 (T_1_ Self-determination). With regard to correlations, they ranged from 0.614 (Self-determination with Positive Enjoyment) to -0.007 (Teacher Controlling Style with Positive Enjoyment). All correlations were significant (*p* < 0.05).

### Latent Profile Analysis

#### Identification of Latent Groups

We compared models between one and four clusters. As can be seen in Table 2, the solution with lower values for AIC and SSA-BIC is the 3 clusters option, which is significantly better than the four clusters option. Based on these results, the three-factor model best represented the dataset.

**Table 2:** Goodness of fit for models with latent groups.

Clusters	AIC	SSA-BIC	PB-LRT (*p*)	% smallest cluster
1	2286.352	2287.394	–	–
2	2231.420	2233.113	0.000	20.000
3	2224.502	2226.846	0.030	14.106
4	2226.495	2229.490	1.000	0.500


*AIC, Akaike Information Criteria; SSA-BIC, Sample size adjusted Bayes Information Criteria; PB-LRT, Parametric Bootstrapped Likelihood Ratio Test.*

#### Description of Latent Groups

**FIGURE 1:**
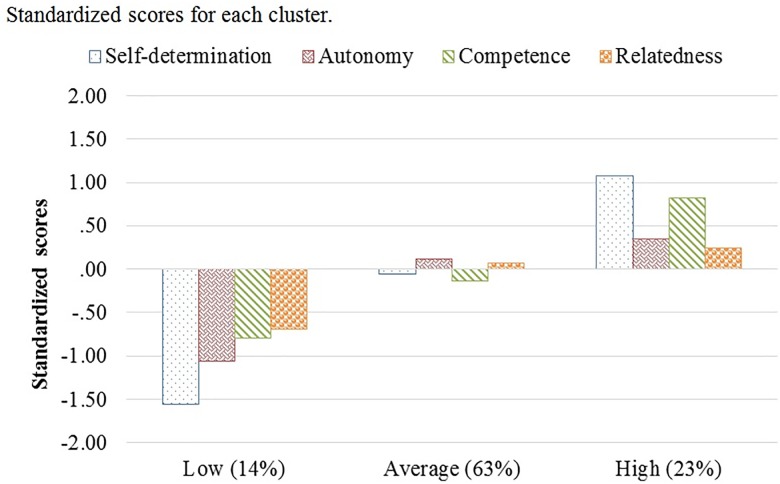
Standardized scores for each cluster.

Figure 1 and Table 3 present the cluster solution. When the changes between the collection 1 and the collection 2 are compared, it is observed that for the first cluster, comprised of 28 students (14%), there were decreases in self-determination and need-fulfillment (low). The second cluster, comprised of 46 (63%), is characterized by no changes in self-determination and need-fulfillment (average). And the third cluster, comprised of 126 (23%), is characterized by increases in self-determination, autonomy, competence, but not in relatedness (high).

**Table 3:** Mean and standard error for variables in each cluster.

		Low	Average	High
		14%	63%	23%
Self-determination	*M*	-1.559	-0.067	1.065
	*SE*	0.222	0.116	0.131
Autonomy	*M*	-1.069	0.111	0.338
	*SE*	0.261	0.095	0.168
Competence	*M*	-0.803	-0.143	0.821
	*SE*	0.292	0.121	0.185
Relatedness	*M*	-0.699	0.065	0.239
	*SE*	0.203	0.107	0.185


*M, Mean; SE, Standard error.*

#### Comparison of Autonomy Support and Positive Enjoyment Across Latent Groups

Subjects in the low cluster had the lowest Autonomy Support (*M* = -1.238) and Positive Enjoyment (*M* = -1.261) variation, subjects in the average cluster showed more Autonomy Support (*M* = 0.0881) and Positive Enjoyment (*M* = -0.024) than subjects in the low cluster, while subjects in the high cluster were characterized by more Positive Enjoyment (*M* = 0.786), but not more Autonomy Support (*M* = 0.493). Results are displayed in Table 4.

**Table 4:** Mean and standard error for autonomy support and positive enjoyment in each cluster.

		Low (1)	Average (2)	High (3)
Autonomy Support	*M*	-1.238^2,3^	0.088^1^	0.493^1^
	*SE*	0.183	0.092	0.189
Enjoyment +	*M*	-1.261^2,3^	-0.024^1,3^	0.786^1,2^
	*SE*	0.184	0.088	0.185


*M, Mean; SE, Standard error. Numbers in superscript refers to groups significantly different (Confidence level = 95%).*

#### Comparison of Control Support and Negative Enjoyment Across Latent Groups

As can be seen in Table 5, subjects in the low cluster had the highest Teacher Controlling style (*M* = 0.773) and Negative Enjoyment variation (*M* = 1.351), subjects in the average cluster showed less Teacher Controlling style (*M* = -0.092) and Negative Enjoyment (*M* = 0.009) than subjects in the low cluster, while subjects in the high cluster were characterized by less Negative Enjoyment (*M* = -0.800), but not less Teacher Controlling style (*M* = -0.214).

**Table 5:** Mean and standard for error teacher controlling style and negative enjoyment in each cluster.

		Low (1)	Average (2)	High (3)
Control Support	*M*	0.773^2,3^	-0.092^1^	-0.214^1^
	*SE*	0.211	0.105	0.163
Enjoyment–	*M*	1.351^2,3^	0.009^1,3^	-0.800^1,2^
	*SE*	0.190	0.084	0.188


*M, Mean; SE, Standard error. Numbers in superscript refers to groups significantly different (Confidence level = 95%).*

#### Comparison of Gender Across Latent Groups

With regard to gender differences, all clusters were significantly different. The low and high cluster were characterized by more females, while more males were observed in the average cluster (Table 6).

**Table 6:** Proportion of males and females in each cluster.

		Low (1)	Average (2)	High (3)
Gender	Male	0.271	0.589	0.000
	Female	0.729	0.411	1.000


## Discussion

The aim of this longitudinal study was twofold: (1) explore different clusters based on autonomy, competence, relatedness and self-determined motivation changes over a 22-months period, and (2) test how these clusters differ on enjoyment and on teacher style (autonomy/control support). The hypothesis presented seeks for the existence of patterns of change and for the association of these patterns with an outcome and a determinant.

### Patterns on Students’ Self-Determined Motivation

Three patterns were identified: decreases in all psychological needs and self-determination; no changes, and increases in self-determination, competence, autonomy but no changes in relatedness. The downward pattern was associated with more Negative Enjoyment and a Controlling Style, while the upward pattern, with more Positive Enjoyment and Autonomy Support. In a similar study, Kimiecik and Horn (2017) observed three different clusters: low, moderate and high, being that the students in the high cluster reported the highest levels of moderate-to-vigorous physical activity and most healthy eating patterns. Kimiecik and Horn’s (2017) findings are significant to understand adolescent self-determined motivation for the participation in health-promoting behavior.

Importantly, those three patterns differed on the outcome (positive and negative enjoyment), and the determinant (autonomy/control support). In a cross-sectional study Wang et al. (2016), observed that students in clusters characterized by the highest levels of autonomous regulation were associated with teachers with a more autonomy-supportive style. Wang et al. (2016) as well as the present study support the SDT since different subtypes of self-determined motivation differentially predict students’ engagement and experience of physical education. It is possible to affirm that it is not only how motivated students are, but in what ways they are motivated, that explain their persistence in physical activities.

The group with low self-determination also showed low fulfillment of the basic psychological needs of autonomy, competence and relatedness with moderate self-determination exhibited moderate fulfillment of these needs. Changes in satisfaction related to basic psychological needs were associated with self-determined motivation, and it is important to examine competence, autonomy and the relationship with others separately, in order to obtain a more complete understanding of the role played by the teaching style, fulfillment of psychological needs and enjoyment of physical activity in adolescents.

Earlier studies gathered evidence of the positive influence of teaching style on autonomy on self-determined motivation, basic psychological needs and satisfaction with physical activity (Lim and Wang, 2009; Cheon et al., 2012; Ntoumanis et al., 2018), since these students are also prone to participate in the proposed tasks, more satisfied with their life and more committed to their activities, in addition to having greater perception of competence (Reeve et al., 2004).

### Patterns on Students’ Enjoyment of Physical Activity

It is important to underscore the relationship between enjoyment and physical activities, given that the sense of fulfillment and pleasure in performing an activity promotes adherence and regularity (Vlachopoulos and Michailidou, 2006). Enjoying physical education classes may increase student commitment to overall physical activities, which increases their desire to remain active outside the school environment, and later become active adults concerned about their health (Ntoumanis, 2005).

There is also an intergroup difference between the sexes. In this study the boys obtained moderate results, while the girls showed both extremes (low and high self-determination). Most of the studies that analyze self-determined motivation to attend physical education classes, considering the students’ sex, found that boys are more intrinsically motivated than girls (Cairney et al., 2012). Moreno-Murcia et al. (2006) observed that the attitudes of female adolescents or adults regarding sport and physical activity follow a more esthetic pattern, while boys display attitudes in relation to team and competitive sports. These differences may be related to attitudes labeled according to sex, stimulating a more competitive attitude in boys, which could cause a feeling of lower competence and less fun during activities.

Educational programs that stimulate the development of self-determined motivation may lead students to habitually engage in physical activities so that they are less likely to discontinue them after the school year ends. Thus, enjoyment in physical education classes results from a more self-determined behavior and fulfillment of the basic psychological needs for competence, autonomy and relationships with others, with an influence of interpersonal teaching style. The present findings support the use of educational strategies that favor autonomy and influence self-determined motivation, thereby promoting commitment to physical activity. In this respect, a number of aspects are important in improving self-determined motivation in physical education classes, such as varying activities, transmitting the feeling of responsibility, enabling student decision making, and recognizing efforts and personal improvement (González-Cutre et al., 2011). As such, it might be interesting for physical education teachers to concentrate on activities that students deem important, interesting and useful as well as stimulate feelings of competence, thereby promoting the perception of success during activities (Gu and Solmon, 2016).

### Limitations

A study limitation was the fact that the authors used the self-determination index, as observed in earlier studies. However, recent research indicates that using a continuum as a general index may dilute the richness of the findings obtained, given that the model can be tested by considering each type of self-determined motivation or separating it into two large categories (autonomous and controlled). Nevertheless, the information collected may help future studies, where it will be important to design interventions with teachers in order to enhance experiences in physical education classes, thereby improving adherence to physical activities.

## Conclusion

In conclusion, more self-determined motivation is related to greater teacher support for autonomy, greater fulfillment of basic psychological needs and increased enjoyment with physical activity. It is important to underscore that the group characterized by high self-determination exhibited high competence values. As such, it is recommended that physical education teachers use a style that supports autonomy, applying strategies that improve student self-determined motivation as well as feelings of pleasure and satisfaction with physical education.

## Author Contributions

JM-M conceptualized the study, supervised and managed the data, drafted and revised the manuscript, approved the manuscript to be published, and agreed to be accountable for all aspects of the work in this manuscript. GF and EB analyzed and managed the data, drafted and revised the manuscript, approved the manuscript to be published, and agreed to be accountable for all aspects of the work in this manuscript. JL and authors conceptualized the study, analyzed and wrote the results, approved the manuscript to be published, and agreed to be accountable for all aspects of the work in this manuscript. RNJ supervised the data, drafted and revised the manuscript, approved the manuscript to be published, and agreed to be accountable for all aspects of the work in this manuscript.

## Conflict of Interest Statement

The authors declare that the research was conducted in the absence of any commercial or financial relationships that could be construed as a potential conflict of interest.
