# Corrigendum: Teachers' Interpersonal Style in Physical Education: Exploring Patterns of Students' Self-Determined Motivation and Enjoyment of Physical Activity in a Longitudinal Study

**DOI:** 10.3389/fpsyg.2021.775300

**Published:** 2021-10-19

**Authors:** Gracielle Fin, Juan Antonio Moreno-Murcia, Jaime León, Elisabeth Baretta, Rudy José Nodari Júnior

**Affiliations:** ^1^Sports Research Center (CID), Universidad Miguel Hernández de Elche, Alicante, Spain; ^2^Department of Physical Education, University of West Santa Catarina, Joaçaba, Brazil; ^3^Department of Education, University of Las Palmas de Gran Canaria, Las Palmas, Spain

**Keywords:** motivation, self-determination, physical activity, enjoyment, adolescents

In the original article, the name of one of the authors was incorrectly spelled in the reference for Soares and Hallal, 2015. Instead of “Sousa and Hallal, 2015” it should be “Soares and Hallal, 2015.” The author's name should also be corrected in the reference list.

The authors apologize for this error and state that this does not change the scientific conclusions of the article in any way. The original article has been updated.

## Publisher's Note

All claims expressed in this article are solely those of the authors and do not necessarily represent those of their affiliated organizations, or those of the publisher, the editors and the reviewers. Any product that may be evaluated in this article, or claim that may be made by its manufacturer, is not guaranteed or endorsed by the publisher.
